# Effects of adherence to treatment for repositioning therapy, physical therapy, and cranial remolding orthoses in infants with cranial deformation

**DOI:** 10.1177/20556683241250310

**Published:** 2024-04-30

**Authors:** Victoria Moses, Caitlin Deville, Susan Simpkins, Jijia Wang, Tally Marlow, Cayman Holley, Shea Briggs, Olivia Sheffer, Amy Payne, Lindsay Pauline, Tristine Lam, Ashton Blasingim, Tiffany Graham

**Affiliations:** 171780 University of Texas Southwestern Medical Center, Dallas, TX, USA

**Keywords:** Orthotics, cranial orthotics, physical therapy, plagiocephaly, torticollis, cranial reshaping, repositioning therapy, cranial remolding

## Abstract

Deformational head shapes are most often treated through repositioning therapy (RT) and/or cranial remolding orthotic (CRO) treatment. However, there is conflicting evidence about the effectiveness of each method, and treatment compliance is suspected to affect treatment effectiveness. This study examines participant adherence with these treatment methods and explores if cranial correction is related to compliance. This study also reviews effects of developmental milestones and explores other potential impacts on compliance. A total of 45 infants with cranial deformation were consented and those with congenital muscular torticollis (CMT) concurrently received physical therapy. Infants were followed from 2 to 12 months of age and initially assigned to RT. Caregivers continued RT until the head shape corrected, caregivers chose to switch to a CRO, or infants turned 12 months of age. All participants were scheduled for a final visit at 12 months of age. Throughout treatment, caregiver surveys were used to examine compliance and developmental milestones. Results show promise for future investigation into the relationship between treatment modalities and adherence with treatment for deformational head shapes. Our findings provide preliminary support that treatment adherence may be linked with treatment success and concurrent enrollment in physical therapy increases patient compliance.

## Introduction

**Figure 1.**
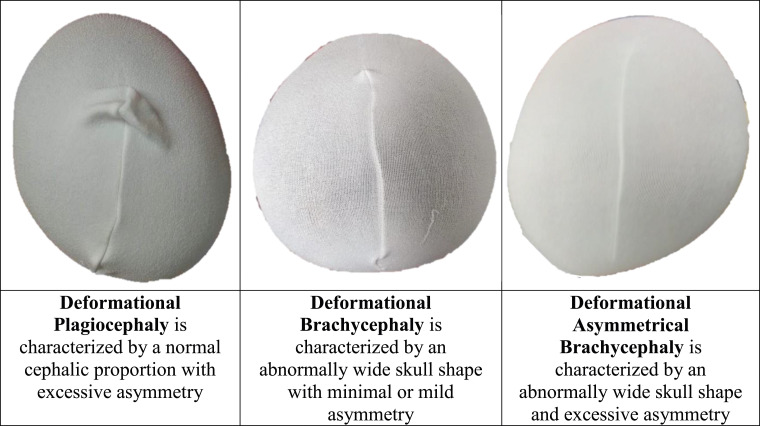
Examples of deformational head shapes and defining characteristics.

The newborn skull is soft, malleable, and vulnerable to deformation by normally occurring forces. Deformation can present as an asymmetry, a proportional abnormality, or a combination of the two (Figure 1). Persistent deformation can be treated either by repositioning therapy (RT) or with a cranial remolding orthosis (CRO).^1^ Infants often begin treatment for deformational head shapes at young ages, before they achieve motor skills like head control, rolling, and sitting independently. Adherence with treatment recommendations such as passive positions or stretches may become more difficult as children gain motor skills. Regardless of treatment method, caregivers must follow treatment protocols for weeks or months, which is often a laborious, tedious, and time-consuming process. Additionally, deformational head shapes often occur concurrently with congenital muscular torticollis (CMT), which should be addressed by physical therapy (PT) and a home stretching regimen.^1^

Multiple publications have observed a need for studies which examine compliance with the treatment of deformational head shapes.^1–3^ Although some studies have compared RT and CRO treatment modalities,^3–5^ they did not report compliance rates or exclude participants with documented non-compliance or those who are lost to follow up, so compliance rates for RT and CRO are currently unknown.^6^

One difficulty with comparing existing studies is the lack of standardization of measurements for the evaluation of deformational head shapes.^7^ The Children’s Healthcare of Atlanta Plagiocephaly Severity Scale (CHOA Scale) is a validated measurement tool for the severity of isolated asymmetry.^5^ There is no general consensus for a severity scale for disproportional head shapes other than the Argenta scale, which uses a visual classification system.^8^

Considering the uncertainty that exists in the literature regarding the best treatment for cranial deformity, there is a call for more studies to examine these treatment options.^1^ Compliance likely affects the efficacy of treatment modalities and therefore should be compared among treatment groups.^3^ This study is intended to examine compliance rates and treatment outcomes during PT, RT, and CRO treatment and review factors which may influence compliance, including developmental milestones.

## Study design

Infants with deformational head shapes were enrolled at 2 months of age (corrected for prematurity, when applicable). PT was provided for infants with CMT and the treatment costs were covered for enrolled participants in an effort to reduce confounding factors such as the influence of untreated CMT or bias based on treatment costs.

Early intervention is considered an important factor for successful CRO treatment.^3,9^ Kluba et al. found that the outcome of treatment was significantly better and treatment duration was shorter for infants who started wearing a CRO prior to 6 months of age.^10^ Other studies have also shown favorable outcomes in this age range,^11^ so all participants decided their final treatment groups at or by 6 months of age. One clinical theory is that RT becomes more difficult to sustain as the infant develops greater strength and control,^12^ so compliance was examined in relationship to developmental milestones.

Private insurance groups often require at least 2 months of attempted RT before CRO intervention is considered.^13^ Therefore, all subjects received 2 months of RT before CRO treatment was offered. This protocol adds to this study’s clinical applicability because care reflects the protocols often seen in clinical practice.

In standard clinical practice, the decision to treat with a CRO is made by the caregiver with advice from the orthotist and physician. The CRO treatment option was explained to caregivers participating in this study with no preference or prompting displayed by the clinician, which reduced the likelihood of bias towards CRO treatment instead of continuing with RT. Since the caregivers had the option to choose their treatment group, it is assumed that treatment compliance was more clinically accurate than in a randomly selected treatment course.

Caregiver surveys were administered at every follow-up appointment. Surveys and the definition of treatment compliance were created by examining prior studies such as surveys used by Seruya et al.,^14^ Pogliani et al.,^15^ and Bialocerkowski et al.^7^

## Methods

Institutional Review Board approval was obtained through University of Texas Southwestern Medical Center (Protocol # STU 032017-036) and two-month-old infants with moderate or severe deformational head shapes were enrolled from January 3, 2019 to November 18, 2021. Study participants began treatment with RT. All caregivers were given the choice of transitioning to CRO treatment at four, five, and six months of age if the deformation had not already corrected with RT. Subjects were followed until 12 months of age and given surveys at every follow up visit (Appendix A-D).

This study uses the validated CHOA severity scale to define the severity of asymmetry based on the cranial vault asymmetry index (CVAI).^5^ The CVAI and cranial index (CI) are often used to quantify changes in cranial shape. CVAI is calculated by taking two diagonals (measured in mm of 30° from the midsagittal line), multiplying the absolute value by 100 and dividing by the largest diagonal (Table 1).^5^ The CI was calculated by taking the percentage of cranial width divided by cranial length (Table 1). All measurements were taken at the greater equator of the skull.

**Table 1.** Cranial vault asymmetry index (CVAI) and cephalic index (CI) calculations where *A* and *B* represent length measurements of the cranial diagonals, taken 30° from midline.^5^

CVAI	$\frac{\left|A-B\right|\cdot 100}{A\;or\;B\;\left(whichever\;is\;largest\right)}$
CI	$\frac{cranial\;width}{cranial\;length}\cdot 100$

At the first visit, infants received a head shape evaluation by a certified orthotist and a CMT screening by a licensed physical therapist. Infants with CMT were concurrently enrolled in physical therapy treatment and a home stretching routine until CMT resolved. Based on the infant’s head shape, the caregivers were educated on RT techniques and seen monthly to monitor head shape changes. The caregivers continued with RT until the head shape was corrected, the infant transitioned to the CRO group, or the infant turned 12 months of age.

**Figure 2.**
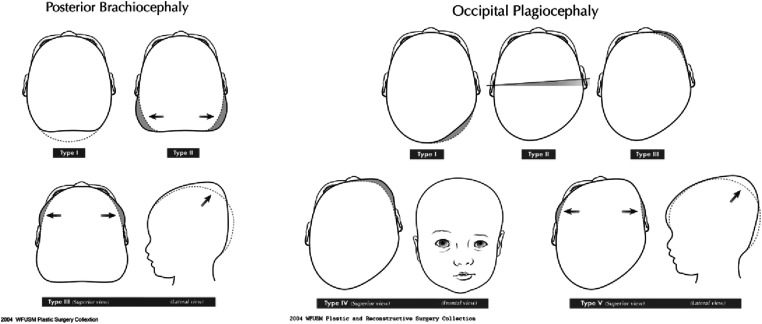
Argenta classification for deformational head shapes (reprinted with permission from the Journal of Craniofacial Surgery).^8^

Craniums were measured with both calipers and a non-invasive scanning system (SmartSoc or STARscanner by Orthomerica Products, Orlando, FL). To be included in the study, infants had either a CI greater than 90% or a CVAI greater than 6.25. The deformation was considered corrected when measurements taken by the orthotist had both a cephalic index below 90% and CVAI below 6.25, and the orthotist determined they were “visually corrected”, which was determined when the deformation was either not clinically apparent or Type I for brachycephaly and not clinically apparent or Type I or II for plagiocephaly according to Argenta’s Classifications (Figure 2). CRO participants had follow up visits every one to four weeks for measurements and adjustments to the CROs.

The presenting severity of the cranial deformation was assessed by caliper measurements of the study orthotist at the time of enrollment. A CVAI between 6.25 and less than 8.75 was considered moderate asymmetry while a CVAI of 8.75 or greater was considered a severe asymmetry. A CI measurement between 90% and 95% was considered a moderate deformation while a CI measurement of 95% or greater was considered a severe deformation. For infants with asymmetrical brachycephaly, the greater of the severity classifications was chosen based on CI and CVAI measurements.

Adherence to RT, PT, and/or CRO treatment was multifactorial and monitored with the use of surveys administered during all follow-up appointments. To be considered compliant with their treatment modality, participants must have attended at least 70% of all required visits and comply with additional treatment-based requirements. To be considered compliant with RT, the caregivers must indicate that they were repositioning ‘always’ or ‘often’ at 90% or more of follow-ups. In the CRO group, the caregivers must indicate that the child wore the CRO 23 hours a day ‘always’ or ‘often’ at 90% or more of follow-ups. In the physical therapy group, the caregivers must indicate that infants received recommended neck stretches at least four times per day. The influence of treatment adherence on achievement of head shape correction was examined to see if trends were present. Additionally, the RT and RT + CRO groups were divided into subgroups based on those who attended and did not attend physical therapy. Compliance of these sub-groups were compared and reported.

The survey questions also included information regarding developmental milestones (Table 2). When the participant achieved a new developmental milestone, treatment compliance was reviewed with the use of a MATLAB code to see if there were any compliance changes at that time. This code also compared RT treatment compliance to CRO treatment compliance (for transitioning infants) and determined if treatment compliance increased, decreased, or remained the same. The compliance questions related to each treatment modality are listed in Table 3.

**Table 2.** Milestone survey questions given to each caregiver at all follow up visits.

My child has independent head control	Yes	No
My child can roll stomach to back	Yes	No
My child can roll back to stomach	Yes	No
My child can sit while propped with hands or small pillows (boppy pillow)	Yes	No
My child can sit independently	Yes	No
My child can crawl	Yes	No
My child can pull to stand	Yes	No
My child can walk (a few steps)	Yes	No
My child can walk (at least 10 ft)	Yes	No
My child attends daycare 3 or more days per week	Yes	No
I am happy with my child’s current head shape	Yes	No

**Table 3.** Compliance survey questions for each treatment group.

RT survey
Every time my infant was laid down for sleep, I confirmed his/her head was in the recommended position Sometimes always often never not applicable
CRO survey
My child is wearing their orthosis (helmet) 23 hours per day Sometimes always often never not applicable
Torticollis PT survey
I Carried out the physical therapist’s recommended stretches to my infant’s neck _________times per day 0 1 2 3 4 >4
In addition to the stretches reported above, a daycare provider or extended family member carried out the physical therapist’s recommended stretches to my infant’s neck ____ times per day 0 1 2 3 4 >4

One of the questions on the RT survey was intended to evaluate how long infants remained in recommended positions after caregivers positioned the infant (“Once positioned, my child remains in that position __: 0-5 min, 5-10 min, 10-30 min, >30 min, or until I move them again”). The percent of RT subjects who were considered compliant and the percent which achieved correction were calculated for each of the different mean positioning time ranges. This was also calculated for the participants which started in RT but later switched to the CRO group.

Caregivers enrolled in physical therapy self-reported the number of times per day the home stretches were performed at each follow up visit. The average number of stretches per day across physical therapy treatment was calculated and reported based on infants who also achieved head shape correction to determine if a correlation was present. All parents were instructed that stretches should be performed four or more times per day.

Participants’ demographic and clinical measurements were compiled in Excel for the statistical analysis. Chi-square test was used to determine if there was a statistically significant difference in gender between RT and CRO groups. Fisher’s exact test was used to compare overall rates of compliance and severity distribution between treatment groups.

Because some infants did not return for their final 12-month visit or were lost to follow up during the course of the study, analyses are stratified by all participants versus participants who completed the study.

## Results

### Summary of treatment modalities for all participants

**Figure 3.**
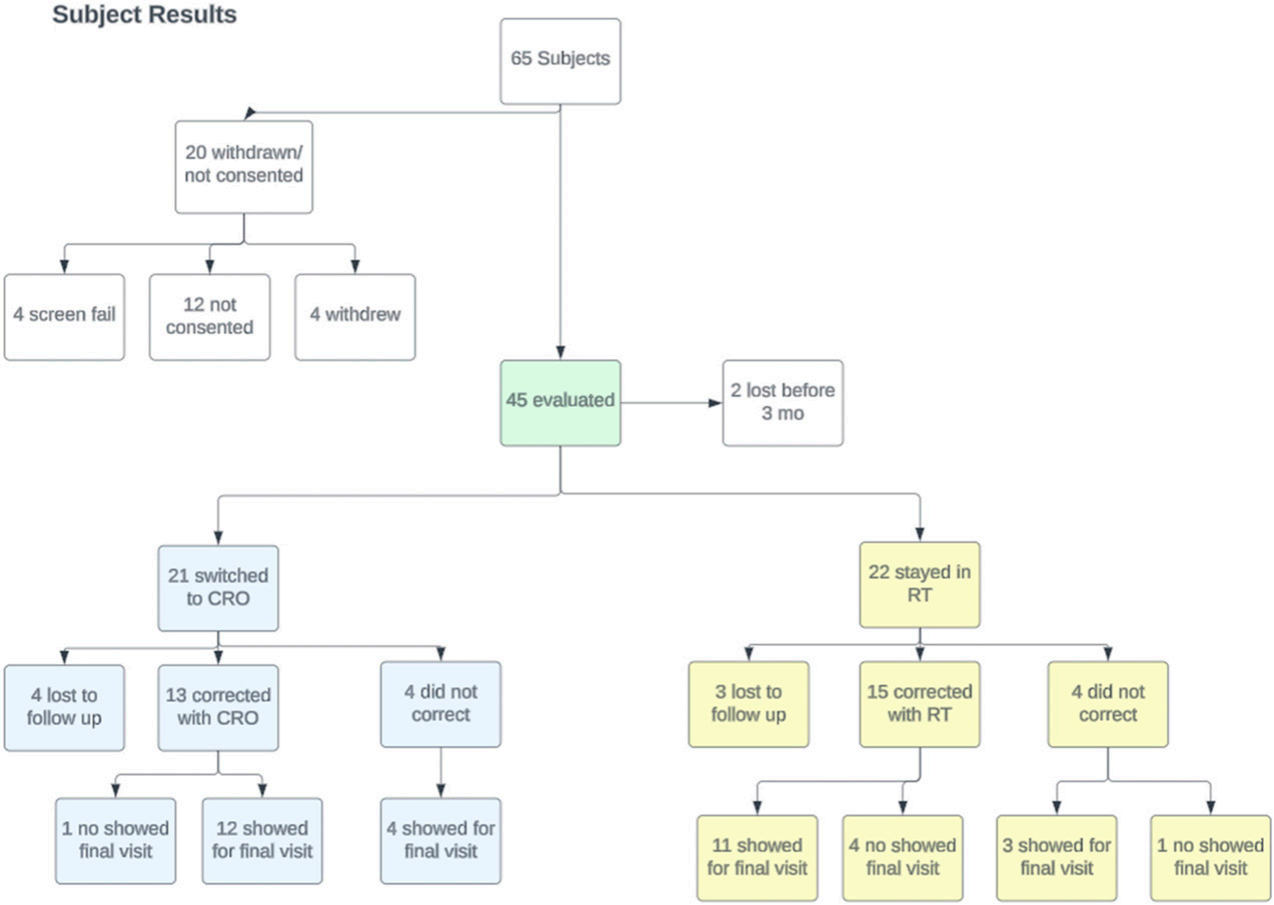
Study participant flow chart. Treatment group and results are depicted. *[RT = Repositioning therapy; CRO = Cranial Remolding Orthosis]*.

A total of 65 infants were referred to the study and 45 of them consented to participate. The excluded subjects chose not to enroll in the study, did not arrive for their evaluation appointment, or had a head shape deformation that was too mild to meet the inclusion criteria. By August 30, 2022, all active subjects were at least 12-month of age. Of the 45 participants, 22 chose to stay in the RT group (48.9%), and 21 switched from the RT to CRO group (46.7%). Two participants were lost to follow up by 3 months of age (4.4%), and therefore were not given an option for CRO and excluded from data analysis since their RT results were unknown. Individual results and compliance summaries are in Appendix E. Figure 3 shows the progression of participants for this study.

Although there were no statistical differences found in the gender distribution between RT and RT + CRO treatment groups (Table 4, *p* = .4164), there was a significant difference in the starting cranial severities in the plagiocephalic group (Table 5). Specifically, in the RT Only group, seven infants started treatment with moderate plagiocephaly and three had severe plagiocephaly, while in the RT + CRO group, one infant began treatment with moderate plagiocephaly and six had severe plagiocephaly (*p* = .0498).

**Table 4.** Demographics of participants’ by gender.

Gender Demographics	Male	Female	Total	*p* value
RT only	12 (54.55%)	10 (45.45%)	22 (51.16%)	0.4164
RT + CRO	14 (66.67%)	7 (33.33%)	21 (48.84%)

**Table 5.** Demographics of participants based on severity of initial head shapes. A significant difference (*) was found in the distribution of moderate versus severe head shapes within the deformational plagiocephaly treatment groups.

Presenting severity of participants based on head shape treatment	Plagiocephaly		Brachycephaly		Asymmetrical brachycephaly		All shapes	
	Moderate	Severe	Moderate	Severe	Moderate	Severe	Moderate	Severe
RT only	7	3	3	3	3	3	13	9
RT + CRO	1	6	1	1	5	7	7	14
Distribution p-value	0.0498*		1.0000		1.0000		0.1292	

### Summary for participants for whose correction outcome is known (*n* = 36)

Of the 36 participants who were followed until their head shape was corrected (or they were 12 months of age and head shape was not yet corrected), 19 completed the study in the RT group (52.8%) and 17 opted for CRO treatment (47.2%). Of the 19 RT subjects, 13 (68.4%) were compliant and one (5.26%) is unknown due to an incomplete survey. Of the 17 CRO subjects, 10 (58.8%) were compliant.

In total, 78.9% (*n* = 15) of the RT subjects achieved clinical correction of their head shape and 76.5% (*n* = 13) of the RT + CRO group achieved clinical correction. Amassing these 36 participants, 28 (77.8%) achieved correction of their head shape during the course of the study, 8 (22.2%) did not achieve full correction. Table 6 shows the breakdown compliance and cranial correction, based on the RT or RT + CRO treatment groups. No statistically significant differences were found in the distribution of infants who did or did not achieve cranial correction based on adherence to the protocol of the treatment groups (when comparing the compliance-based correction rates of participants in the RT Only group, *p* = .2839; when comparing compliance-based correction rates for the RT + CRO group, *p* = .25). Of note, one of the infants in “RT Only” group reached correction, but their compliance was unknown due to incomplete survey.

**Table 6.** Distribution of cranial correction and correction rates based on adherence to protocols within the treatment groups (no statistical differences found in correction rates based on compliance).

Influence of treatment compliance on head shape correction (*n* = 35)				
Treatment group	RT only		RT + CRO	
Compliance	RT compliant	RT non-compliant	CRO compliant	CRO non-compliant
Corrected	11	3	9	4
Did not correct	2	2	1	3
Correction rate	84.62%	60.00%	90.00%	57.14%

### Summary for all participants (*n* = 43)

Five subjects did not return at 12 months of age for their final assessment, but did continue treatment until resolution of their head shape. Of the 43 participants who were followed beyond 3 months of age, 22 (51.16%) remained in the repositioning group. Of these 22 participants, 13 were compliant (59.09%), 5 were not compliant (22.73%), and four were lost to follow-up or lacked a survey answer (18.18%). Among the CRO group, 10 participants (47.62%) were compliant, seven (33.33%) were non-compliant, and four (19.05%) were lost to follow-up. Appendix E details individual participant compliance and correction. RT + CRO Infants who were concurrently enrolled in physical therapy were statistically more compliant (Table 7, *p* = .0176); however, no statistical differences were found in participant compliance with RT based on concurrent PT enrollment (*p* = 1.000). In total, 68.18% (*n* = 15) of the RT subjects achieved clinical correction of their head shape and 61.9% (*n* = 13) of the CRO group achieved clinical correction.

**Table 7.** Treatment compliance based on treatment group. A statistical difference (*) was found in the compliance of the RT+CRO group based on concurrent enrollment in PT.

Caregiver-reported treatment compliance				
Treatment group	# infants	# Compliant	% compliant	*p*-value
RT + PT	21^a^	14^b^	66.57	1.0000
RT only	7	5	71.43	
All RT subjects^a^	28	19	67.86	
RT + CRO + PT	13	11	84.62	0.0176*
RT + CRO only	8^a^	2	25.00	
All RT + CRO subjects	21	13	61.90	

^a^Includes six infants who finished with PT before receiving their CRO.

^b^One infant’s compliance is unknown due to a lack of survey answer and not reported.

The following compliance changes seemed to be associated with survey response changes (response changes detailed in Appendix F): One participant became compliant with RT when they gained independent head control. One participant became compliant with RT and one participant became compliant with CRO treatments when they were able to roll stomach to back while three participants became noncompliant with RT when they were able to roll stomach to back. Two RT and one CRO participant became compliant when they were able to roll back to stomach, and four participants in RT became treatment non-compliant when able to roll back to stomach. The remaining milestone questions asked in the survey showed no patterns in relation to treatment compliance.

A change in daycare attendance resulted in two RT participants becoming non-compliant; however, one was when the child started attending day care and one was when the child stopped attending daycare. No changes in treatment compliance were noted in the CRO group based on changes in daycare attendance.

Regarding the amount of time each infant was able to spend in a repositioned position before moving (42 repositioning respondents, which includes those who transitioned to CRO), on average, 7.14% were able to position for 0–5 min a day (66.7% of which were considered treatment compliant and overall 66.7% achieved correction). 50.00% were able to position for 5–10 min a day (76.19% of which were considered treatment compliant and overall 57.14% achieved correction). 28.57% were able to position for 10–30 min a day (75% of which were considered treatment compliant and overall 75% achieved correction). 14.29% were able to position for >30 min a day (66.67% of which were considered treatment compliant and overall 66.67% achieved correction).

Of the 21 participants in the RT + CRO group, 42.86% were able to stay in the repositioned position for 5–10 min. Of these, 44.44% achieved correction in CRO and 44.44% were lost to follow-up. After moving to CRO, 66.67% of RT + CRO participants were able to position for 10–30 min at a time. Of these, 87.50% achieved correction. After moving to CRO, 66.67% of RT + CRO participants were able to position for >30 min at a time. Of these, 50% achieved correction (Table 8).

**Table 8.** Descriptive table of infant’s compliance and head shape correction rate based on caregiver’s repositioning efforts, stratified by treatment group. *[RT = repositioning therapy; CRO = Cranial Remolding Orthosis; n/a = not applicable]*.

Head shape correction rates based on Infant’s compliance with repositioning efforts										
Once positioned, my child remains in that position (on average)	% Of RT participants (*n* = 42)	% Of RT participants who transitioned to CRO (*n* = 21)	Compliant with RT		Lost to follow up		Corrected		Not corrected	
			RT only (*n* = 21)	RT + CRO (*n* = 21)	RT only (*n* = 21)	RT + CRO (*n* = 21)	RT only (*n* = 21)	RT + CRO (*n* = 21)	RT only (*n* = 21)	RT + CRO (*n* = 21)
0–5 min	7.14	0.00	66.67%	0.00%	33.33%	n/a	66.67%	n/a	0.00%	n/a
5–10 min	50.00	42.86	66.67%	88.89%	16.67%	44.44%	66.67%	44.44%	16.67%	11.11%
10–30 min	28.57	66.67	75.00%	75.00%	0.00%	0.00%	50.00%	87.50%	50.00%	12.50%
>30 min	14.29	66.67	50.00%	75.00%	0.00%	0.00%	100.00%	50.00%	0.00%	50.00%
Until I move them	0.00	0.00	n/a	0.00%	n/a	n/a	n/a	n/a	n/a	n/a

A total of 34 infants were concurrently enrolled in physical therapy due to the presence of CMT and 25 of these infants had complete survey data regarding their home stretching exercises (Table 9). Although all caregivers were instructed to perform the home stretching four or more times daily, of the 25 respondents, only 28.00% of infants achieved this goal across the duration of physical therapy treatment. Table 9 divides infants enrolled in physical therapy based on their head shape severity at two months of age based on the average number of daily neck stretches performed and their eventual resolution or non-resolution of the cranial deformation. Caregivers for seven infants enrolled in PT reported performing stretches at least four times daily, indicating compliance with the recommended stretching program. From this group, five infants achieved eventual cranial correction, one did not achieve correction, and one had unknown results. In comparison, caregivers for 18 infants reported performing stretches less than four times daily, indicating lack of compliance with the recommended stretching program. From this group, 11 achieved cranial correction, five did not achieve correction, and two were lost to follow up after PT so final head shape was unknown. The correction rate differences were not statistically significant between groups, although there was a greater correction rate in the group who performed stretches four or more times per day (71.4% versus 61.11%).

**Table 9.** Descriptive table of the rates of cranial deformation correction achieved, based on compliance with the physical therapy home stretching [*s = number of daily stretches*].

Head shape correction rates for infants undergoing home stretches and physical therapy								
Average number of daily stretches	# Infants with moderate deformation (*n* = 9)				# Infants with severe deformation (*n* = 16)			
	Head shape corrected	Head shape not corrected	Lost to follow-up after PT	Treatment compliant with RT/CRO	Head shape corrected	Head shape not corrected	Lost to follow-up after PT	Treatment compliant with RT/CRO
0 ≤ s < 1	—	—	—	—	1 (100%)	—	—	1 (100%)
1 ≤ s < 2	1 (100%)		—	1 (100%)	—	—	—	—
2 ≤ s < 3	—	1 (100%)	—	1 (100%)	3 (60%)	1 (20%)	1 (20%)	2 (40%)
3 ≤ s < 4	2 (66.67%)	1 (33.33%)	—	2 (66.67%)	4 (57.14%)	2 (28.57%)	1 (14.29%)	5 (71.43%)
4 ≤ s	2 (50%)	1 (25%)	1 (25%)	3 (75%)	3 (100%)	—	—	2 (66.67%)

## Discussion

### Caregiver surveys

Results are based on parental survey responses for particularly worded questions. Comprehension of the question, understanding of the intended milestone, subjective interpretations, and language barriers could have impacted responses. Additionally, factors such as which caretaker was present for each appointment or the presence of acquiescence bias, question order bias, or survivorship bias may have impacted results.

The caregivers were informed that their responses would not influence their clinical care, and to answer all questions honestly; however, answers may be impacted by direct observation, and factors motivating acquiescence bias could include appearing as an attentive, responsible, compliant caretaker.^10^ Additionally, questions concerning developmental milestones may be points of pride for the caretakers and if they mistakenly answered “yes” on one visit, they may be hesitant to correct their answers on future surveys. That being said, it was noted for some participants, a milestone had been marked as achieved, then marked as not achieved on a consecutive survey. This was interpreted by the research team as a “false” milestone achievement, possibly caused by the infant performing the milestone once or twice, but not yet consistently. These “false” milestone responses were manually corrected to indicate the child had not yet achieved the milestone.

### Sample size and significance

Since this pilot study had 43 participants who participated beyond 3 months of age, data analysis is limited in its scope. Although statistical significance was examined, clinical significance was not defined or reported. As discussed in the following sections, the findings of this study correspond to clinical observations and relay promising trends in the sample data collected. A larger, multi-site trial is needed to further explore statistical differences between treatment groups.

Statistically significant differences were found in the distribution of severe and moderate head shapes between the RT and the RT + CRO treatment groups for infants with deformational plagiocepahly (Table 5). Infants in the RT + CRO group who were concurrently enrolled in PT were statistically more compliant to their treatment protocol of wearing the CRO 23 hours per day (Table 7).

Compliant infants were observed to have higher correction rates than those who were found to be non-compliant with their respective treatment protocols (Table 6). A 25%–33% better rate of correction is clinically significant (84.62% of RT infants who adhered to the protocols achieved cranial correction while only 60% of non-compliant RT infants achieved cranial correction; 90% of compliant infants in the RT + CRO group achieved cranial correction while 57.14% of non-compliant infants achieved cranial correction.) A post-hoc analysis was performed to estimate the size of the clinical trial needed to potentially find statistical significance in these numbers. With 80% power, we likely need 98 patients in each group. A larger study and further investigation into this trend is warranted.

### Demographic breakdown

This study and some past studies have observed a larger male population of infants with cranial deformation,^16^ but statistical significance was not found in gender distribution of this cohort.

It has been clinically observed in this and other studies that infants with more severe deformational head shapes seemed to transition to CRO treatment sooner and more often than infants with less severe deformations.^17,18^ This study may also have been impacted by the nature of referrals and compensation. Participants were referred to the investigators by pediatricians who referred based on their understanding of the selection criteria (moderate to severe cranial deformation), which could introduce selection bias. Additionally, since enrolled infants were provided RT, PT (if indicated), and a CRO at no cost to the participant, this may have influenced referrals of particular patient populations. However, providing treatment at no cost was both an incentive and metric to increase participation. It offers the opportunity for treatment to participants whose insurance policies do not cover CRO treatment and provides earlier access to treatment than some insurance policies currently allow.

In this study, the RT group did have a greater number of moderate infants than severe infants and the RT + CRO group had twice as many severe infants as moderate infants (Table 5). This is likely why the correction rates in the RT group were observed to be slightly higher than those in the RT + CRO group (78.9% versus 76.5%, respectively); however, three infants were lost to follow up in the RT group and four infants were lost to follow up in the CRO group, which could influence these numbers.

For the 35 infants who completed the study protocol (and whose compliance was known), the correction rates between treatment groups were not found to be statistically different (*p* = 1.0000), which is clinically significant. In this case, infants with more severe deformations (RT + CRO group) are achieving clinically similar correction rates to infants with more moderate deformations (RT group). The RT + CRO group reported lower adherence to the treatment protocol than the RT group (58.8% versus 72.2%). Based on the results of Table 6, improving compliance leads to higher correction rates. For example, compliant patients in the RT + CRO group achieved clinical correction 90% of the time in the observed cohort. Therefore anything which improves adherence to treatment protocols should be encouraged by treating clinicians in order to achieve more optimal treatment outcomes.

This study enrolled all participants at 2 months of age and concluded when they turned 12 months of age; however, multiple caregivers in the RT + CRO group opted to continue CRO use beyond the timeline of the study because they were continuing to see improvements to their child’s cranial shape.

### Compliance relative to treatment group

The reported treatment compliance is highly dependent upon how compliance was defined. Although all participants must have attended at least 70% of scheduled visits to be considered compliant, the treatment modalities had differing additional compliance criteria. Specifically, PT compliance was determined by the clinical recommendation that infants undergo home stretching (on average) at least four times per day^19^ while CRO compliance was determined if the infants were wearing their CRO “always” or “often” 23 hours per day at 90% of the follow up visits. The RT compliance determination was based on a question which only asked about head positioning when the infant was laid to sleep. Other factors were recorded (such as daily tummy time), but not used in this preliminary analysis for the definition of treatment compliance. Further analyses are needed to see which survey questions were statistically correlated to treatment outcomes in order to better define treatment compliance in each treatment modality.

As shown in Table 7, infants in the RT + CRO group who participated in PT had statistically better adherence to treatment (*p* = .0176). Infants with CMT in either group who underwent an average of four or more stretches per day throughout treatment corrected 71.43% of the time (five out of seven corrected, one outcome unknown) while infants who underwent fewer than four stretches per day corrected 61.16% of the time (11 out of 18 corrected, two unknown). Further investigation should be done to examine if additional contact with health care providers directly increased treatment adherence (and therefore cranial correction) or if potentially, reduced muscle forces on the skull from improved range of motion contributed to better head shape correction.

### Influence of developmental milestones on treatment compliance

Gaining independent head control may make compliance with RT more challenging since the child may prefer laying on one side and Bialocerkowski et al. noted that treatment becomes more difficult when a participant has a strong head side preference.^7^ Historical clinical observations have been that once a child is able to roll, compliance often decreases in RT. In our study, acquisition of rolling stomach to back or back to stomach decreased compliance for seven participants in the RT group. If infants become able to move themselves independently out of RT positions, it seems logical that compliance with RT would be more difficult.

Infants undergoing CRO treatment had fewer compliance changes in response to milestones than those in RT. During CRO treatment, five out of the nine milestone questions had no change in compliance when the child achieved the developmental milestone. Additionally, three out of the four milestone compliance changes were all due to the same participant switching compliance.

Survey responses regarding infants undergoing physical therapy to address CMT treatment were analyzed to determine if there was change in CMT treatment compliance in relation to developmental milestones. Due to the small sample size and survey inconsistences, there no statistically significant changes were found for CMT treatment compliance related to the development of a motor milestone.

Further research should be completed to investigate if developmental milestones impact treatment compliance in a large sample size.

### Daycare attendance

It was hypothesized that participants who attended daycare more frequently would be less treatment compliant since the family is not present to implement the treatment protocol, especially in the RT group. However, in this cohort, no significant differences were found.

### Infant compliance with repositioning

For the RT-only group, all of the participants which were able to remain in a repositioned state for 30 min or more once their child was laid to sleep achieved cranial correction (Table 8) and the two who did not correct who were able to maintain their position for 10–30 min both had severe deformational brachycephaly.

When examining the cranial correction in the group which transitioned to a CRO (Table 8), the infant who did not achieve cranial correction which was previously able to stay in a repositioned pose for 5–10 min, had a severe cranial deformation. It should be noted that the patient did achieve clinically and visually significant correction, but did not achieve the measurements needed to be considered “corrected” within the timeline of the study. This infant’s caregivers elected to continue treatment beyond 12 months of age (outside of the study). For participants in the transition group who were previously able to position for 10–30 min, one participant did not achieve cranial correction in the CRO and was not compliant with the CRO. The two infants who did not achieve cranial correction within the CRO which were previously able to position for >30 min per day, were not compliant with the CRO. All other participants achieved correction or were lost to follow-up. A future study should explore the infant’s adherence to repositioning protocols to as this appears to be a clinically significant factor in the success of repositioning treatment and may help predict success of CRO treatment for infants to transition to CROs.

### Future study improvements

The use of pressure sensors or temperature sensors (such as the iButton), could be implemented to obtain more reliable data on CRO treatment adherence rather than self-reported surveys. Adding compensation for the completion of the study may encourage more follow through from the participant’s family.

The definitions of treatment compliance could be reconsidered. For example, instead of defining RT compliance as laying the child on the correct or alternating head side each time they went to sleep, the study could track amount of time spent in a repositioned position. If alternative definitions of compliance were measured, future studies may gain insight into parental trends or patient compliance.

## Conclusion

Treatment adherence can be impacted by various factors, including treatment modality. Several of the reported results do correlate to clinical observations and could be clinically significant, particularly considering the influence of rolling on RT treatment compliance and the influence concurrent physical therapy seems to have on CRO treatment adherence. Future larger studies should examine alternative definitions of compliance, more objectively monitor compliance, and perhaps include a more objective assessment of developmental milestone achievement.

Infants with increased treatment adherence to their respective treatment modality (RT, CRO treatment, and PT) were observed to have a higher incidence of cranial correction. Infants enrolled in concurrent PT generally were found to have greater adherence with CRO treatment which was statistically significant. Clinicians seeing infants with deformational head shapes should encourage adherence to the child’s respective treatment protocol whenever possible for more optimal treatment outcomes. As a pilot study, these results show promise for future investigation into the relationship between treatment modalities and compliance in the treatment of deformational head shapes.

## Supplemental Material

Supplemental Material - Effects of adherence to treatment for repositioning therapy, physical therapy, and cranial remolding orthoses in infants with cranial deformation Supplemental Material for Effects of adherence to treatment for repositioning therapy, physical therapy, and cranial remolding orthoses in infants with cranial deformation by Victoria Moses, Caitlin Deville, Susan Simpkins, Jijia Wang, Tally Marlow, Cayman Holley, Shea Briggs, Olivia Sheffer, Amy Payne, Lindsay Pauline, Tristine Lam, Ashton Blasingim and Tiffany Graham in Journal of Rehabilitation and Assistive Technologies Engineering.
