# Supplementary material for: Effects of adherence to treatment for repositioning therapy, physical therapy, and cranial remolding orthoses in infants with cranial deformation
Source: J Rehabil Assist Technol Eng. 2024 Apr 30;11:20556683241250310. doi: 10.1177/20556683241250310 (PMC11062220; doi:10.1177/20556683241250310)
Supplement: Supplemental Material - Effects of adherence to treatment for repositioning therapy, physical therapy, and cranial remolding orthoses in infants with cranial deformation [file sj-pdf-1-jrt-10.1177_20556683241250310.pdf]

Appendix A: Survey Questions for Repositioning Families

Questions for Repositioning Families

Please circle the most appropriate answer since your child's last visit to the UT Southwestern Clinic

I understand how to position my child to try to correct his/her head shape	YES	NO
My child is swaddled for sleep	YES	NO
My child moves around during sleep (turns over, spins, etc)	YES	NO
My child has independent head control	YES	NO
My child can roll stomach to back	YES	NO
My child can roll back to stomach	YES	NO
My child can sit while propped with hands or small pillows (boppy pillow)	YES	NO
My child can sit independently	YES	NO
My child can crawl	YES	NO
My child can pull to stand	YES	NO
My child can walk (A few steps)	YES	NO
My child can walk (at least 10 feet)	YES	NO
My child attends daycare 3 or more days per week.	YES	NO
I have noticed improvement in my child's head shape	YES	NO
I am happy with my child's current head shape	YES	NO
My child has been identified to need developmental therapy	YES	NO
---If yes what therapy?		

Please circle the most appropriate answer since your child's last visit to the UT Southwestern Clinic

Every time my infant way laid down for sleep, I confirmed his/her head was in the recommended position.
ALWAYS OFTEN SOMETIMES RARELY NEVER NOT APPLICABLE
(If told to alternate sides) I changed the sleep position between left and right sides every time I laid my child to sleep.
ALWAYS OFTEN SOMETIMES RARELY NEVER NOT APPLICABLE
(If told neck is tight) I hold my child in the recommended football position to stretch their neck every day, multiple times per day.
ALWAYS OFTEN SOMETIMES RARELY NEVER NOT APPLICABLE
(If getting physical therapy for a tight neck) I do the physical therapist recommended stretches daily, multiple times per day (i.e. 2 or more times daily).
ALWAYS OFTEN SOMETIMES RARELY NEVER NOT APPLICABLE
(if told neck is tight) I try to direct my child to look/turn toward the non-preferred side.
ALWAYS OFTEN SOMETIMES RARELY NEVER NOT APPLICABLE
My child is happy to do tummy time activities.
ALWAYS OFTEN SOMETIMES RARELY NEVER NOT APPLICABLE
My child does tummy time at least ______________ : (N/A if your child is walking)
5 minutes/day 20 minutes/day 40 minutes/day 1 hour/day 2 hours/day 3+ hours/day N/A
Once positioned, my child remains in that position ____________:
0-5 minutes 5-10 minutes 10-30 minutes >30 minutes until I move them again
My child spends approximately ________ in a car seat daily:
0-20 minutes 20-40 minutes 40-60 minutes 1-2 hours 2-3 hours 3+ hours

Appendix B: Survey Questions for CRO Families

Questions for CRO Families

Please circle the most appropriate answer since your child's last visit to the UT Southwestern Clinic

My child is swaddled for sleep	YES	NO
My child moves around during sleep (turns over, spins, etc)	YES	NO
My child has independent head control	YES	NO
My child can roll stomach to back	YES	NO
My child can roll back to stomach	YES	NO
My child can sit while propped with hands or small pillows (boppy pillow)	YES	NO
My child can sit independently	YES	NO
My child can crawl	YES	NO
My child can pull to stand	YES	NO
My child can walk (A few steps)	YES	NO
My child can walk (at least 10 feet)	YES	NO
My child attends daycare 3 or more days per week.	YES	NO
I have noticed improvement in my child's head shape	YES	NO
I have noticed a worsening in my child's head shape	YES	NO
I am happy with my child's current head shape	YES	NO
My child can take the orthosis off by himself/herself	YES	NO

Please circle the most appropriate answer since your child's last visit to the UT Southwestern Clinic

My child is wearing their orthosis (helmet) 23 hours per day. (N/A if 1st week in orthosis)
ALWAYS OFTEN SOMETIMES RARELY NEVER NOT APPLICABLE
The orthosis (helmet) was off for _________ days total since the last visit due to (circle one):
SKIN FEVER OTHER:________________
My child tolerates the orthosis.
ALWAYS OFTEN SOMETIMES RARELY NEVER NOT APPLICABLE
(If getting physical therapy for a tight neck) I do the physical therapist recommended stretches daily, multiple times per day (i.e. 2 or more times daily).
ALWAYS OFTEN SOMETIMES RARELY NEVER NOT APPLICABLE
(If told neck is tight) I position my child to turn toward their non-preferred side for every diaper change and feeding.
ALWAYS OFTEN SOMETIMES RARELY NEVER NOT APPLICABLE
(If told neck is tight) I try to direct my child to look/turn toward the non-preferred side.
ALWAYS OFTEN SOMETIMES RARELY NEVER NOT APPLICABLE
I clean the orthosis (helmet) at least 1 time daily.
ALWAYS OFTEN SOMETIMES RARELY NEVER NOT APPLICABLE

Appendix C: Survey Questions for Families of Children Receiving Physical Therapy for Torticollis

Questions for Families of Children Receiving Physical Therapy for Torticollis

Please circle the most appropriate answer since your child's last visit to the UT Southwestern Clinic

I feel confident that I understand how to carry out the physical therapist's recommended stretches to my infant's neck.
ALWAYS OFTEN SOMETIMES RARELY NEVER
I carried out the physical therapist's recommended stretches to my infant's neck _________ times per day.
0 1 2 3 4 > 4
In addition to the stretches reported above, a daycare provider or extended family member carried out the physical therapist's recommended stretches to my infant's neck ____ times per day.
0 1 2 3 4 > 4
I followed the physical therapist's recommendations for home modifications to encourage my infant to turn their head toward the non-preferred side.
ALWAYS OFTEN SOMETIMES RARELY NEVER NOT APPLICABLE
I used the football carry position to stretch my infant's neck _______ times per day.
0 1 2 3 4 > 4
My child does tummy time at least ________ minutes per day
0 5 20 40 60 > 60
My child is happy to do tummy time activities.
ALWAYS OFTEN SOMETIMES RARELY NEVER NOT APPLICABLE
My child spends approximately ________ minutes per day in a supported sitting position.
0-15 15 -30 30-40 40- 60 > 60

My child attends daycare 3 or more days per week.
YES NO
Please comment on any factors that prevented you from following your infants home program since your last visit to the UT Southwestern clinic:

Appendix D: Survey Questions for Final 12 month visit

Final Survey 12 Months of Age

Please circle the most appropriate answer since your child's last visit to the UT Southwestern Clinic

Per my pediatrician, my child is meeting/exceeding their developmental milestones.	YES	NO
During the course of this study, my child had developmental therapy	YES	NO
a. If yes, what therapy?		
b. Treatment started at _______ (child age or date) and ended at ___________.		
(If neck tightness was identified) My child's neck is still tight.	YES	NO
a. If no, when did you discontinue stretches or therapy?		
I am happy with my child's current head shape.	YES	NO
I have noticed improvement in my child's head shape over the course of this study.	YES	NO
If it were possible to have a "do over", would you still choose the same treatment option for your child?	YES	NO
Would you be willing to either have your child measured again or take a survey in the future?	YES	NO

Appendix E: Summary of individual participant demographics, compliance and correction (n=43). Green text indicates the infant achieved clinical correction of their cranial deformation. Red text indicates they did not achieve clinical correction. Black text indicates an unknown result of their final head shape due to being lost to follow up. *[DP = Deformational Plagiocephaly, DAB = Deformational Asymmetrical Brachycephaly, DB = Deformational Brachycephaly]*

**Participant Summary**										
**#**	**Sex**	**Severity**	**Head Shape**	**Corrected in RT?**	**RT Compliant?**	**CRO Transition Age**	**Corrected in CRO?**	**CRO Compliant?**	**Age at Clinical Correction**	**PT for Torticollis?**
1	M	Moderate	DP	N, moved to CRO	N	6 months	Y	N	8 months	N
2	M	Severe	DAB	N, moved to CRO	Y	4 months	Lost to Follow-up	N	Unknown	Y
3	F	Severe	DB	N	N	N/A	N/A	N/A	N/A	N
4	M	Moderate	DAB	N, moved to CRO	Y	4 months	Lost to Follow-up	Y	Unknown	Y
5	F	Moderate	DP	N	N	N/A	N/A	N/A	N/A	Y
6	M	Moderate	DAB	Y	Y	N/A	N/A	N/A	11 months	Y
7	M	Severe	DP	N, moved to CRO	Y	5 months	Lost to Follow-up	Y	Unknown	Y
8	M	Severe	DAB	N, moved to CRO	Y	4 months	Y	Y	9 months	Y
9	M	Moderate	DB	Y	Y	N/A	N/A	N/A	6 months	Y
10	M	Severe	DB	N	Y	N/A	N/A	N/A	N/A	Y
11	M	Severe	DAB	N, moved to CRO	Y	4 months	Y	N	8 months	Y
12	M	Severe	DAB	N, moved to CRO	Y	4 months	Y	N	9 months	Y
14	M	Moderate	DAB	N, moved to CRO	Y	4 months	Lost to Follow-up	N	Unknown	Y
16*	M	Severe	DP	N	Y	N/A	N/A	N/A	N/A	Y
17	M	Moderate	DB	N, moved to CRO	N	6 months	N	Y	N/A	Y
18	M	Moderate	DP	Y	Y	N/A	N/A	N/A	7 months	Y
19	F	Severe	DP	N, moved to CRO	Y	5 months	N	N	N/A	Y
20	F	Moderate	DAB	Y	Y	N/A	N/A	N/A	7 months	Y
23	M	Moderate	DP	Y	unknown	N/A	N/A	N/A	4 months	Y
25	F	Moderate	DAB	N, moved to CRO	Y	6 months	N	N	N/A	Y
27	F	Severe	DAB	N, moved to CRO	N	5 months	N	N	N/A	N
28	M	Severe	DB	Lost to Follow-up	N	N/A	N/A	N/A	Unknown	Y

30	M	Severe	DP	N, moved to CRO	Y	4 months	Y	Y	9 months	Y
31	M	Moderate	DP	Y	Y	N/A	N/A	N/A	6 months	N
32	F	Moderate	DAB	Y	N	N/A	N/A	N/A	10 months	Y
33	F	Moderate	DB	Y	Y	N/A	N/A	N/A	6 months	Y
38	F	Moderate	DB	Y	Y	N/A	N/A	N/A	4 months	N
41	M	Severe	DP	Y	Y	N/A	N/A	N/A	5 months	Y
42	F	Severe	DP	N, moved to CRO	Y	5 months	Y	Y	8 months	Y
43	F	Severe	DAB	Y	N	N/A	N/A	N/A	5 months	Y
44	F	Moderate	DP	Lost to Follow-up	Y	N/A	N/A	N/A	Unknown	Y
46	F	Severe	DAB	Y	N	N/A	N/A	N/A	8 months	Y
48	M	Moderate	DAB	N, moved to CRO	N	6 months	Y	N	11 months	Y
49	M	Severe	DB	N, moved to CRO	Y	4 months	Y	Y	10 months	Y
50	M	Severe	DP	Lost to Follow-up	N	N/A	N/A	N/A	Unknown	N
51	F	Severe	DAB	N, moved to CRO	Y	4 months	Y	Y	10 months	Y
52	F	Moderate	DAB	N, moved to CRO	Y	4 months	Y	Y	10 months	Y
54	M	Moderate	DP	Y	Y	N/A	N/A	N/A	6 months	N
57	M	Severe	DAB	N, moved to CRO	Y	6 months	Y	Y	10 months	Y
58	F	Severe	DAB	Y	Y	N/A	N/A	N/A	12 months	N
59	M	Severe	DP	N, moved to CRO	Y	4 months	Y	Y	8 months	Y
60	M	Moderate	DP	Y	Y	N/A	N/A	N/A	4 months	N
61	F	Severe	DP	N, moved to CRO	Y	5 months	Y	Y	8 months	Y

* Patient #16 started with RT, transitioned to CRO, but did not wear the CRO and was therefore returned to the RT only group.

Appendix F. RT, CRO, & PT data comparing each developmental milestone to treatment compliance.

**RT & CRO data comparing developmental milestone to treatment compliance (Q17)**		
My child has independent head control		
**Group**	**Became Treatment compliant**	**Became Treatment Non-compliant**
RT	1	-
CRO	-	-
PT-RT	-	-
PT - CRO	-	-
My child can roll stomach to back		
**Group**	**Became Treatment compliant**	**Became Treatment Non-compliant**
RT	1	3
CRO	1	-
PT - RT	-	-
PT - CRO	-	-
My child can roll back to stomach		
**Group**	**Became Treatment compliant**	**Became Treatment Non-compliant**
RT	2	4
CRO	1	-
PT-RT	-	-
PT - CRO	-	-
My child can sit while propped with hands or small pillows (boppy pillow)		
**Group**	**Became Treatment compliant**	**Became Treatment Non-compliant**
RT	1	-
CRO	1	-
PT - RT	-	-
PT - CRO	-	-
My child can sit independently		
**Group**	**Became Treatment compliant**	**Became Treatment Non-compliant**
RT	2	3
CRO	-	-
PT-RT	1	1
PT - CRO	1	-
My child can crawl		
**Group**	**Became Treatment compliant**	**Became Treatment Non-compliant**
RT	2	1
CRO	-	-
PT - RT	-	-
PT - CRO	-	-
My child can pull to stand		
**Group**	**Became Treatment compliant**	**Became Treatment Non-compliant**
RT	3	1
CRO	-	1

PT - RT	-	-
PT - CRO	-	1
My child can walk (a few steps)		
**Group**	**Became Treatment compliant**	**Became Treatment Non-compliant**
RT	1	1
CRO	-	-
PT - RT	-	-
PT - CRO	-	1
My child can walk (at least 10ft)		
**Group**	**Became Treatment compliant**	**Became Treatment Non-compliant**
RT	-	1
CRO	-	-
PT - RT	-	-
PT - CRO	-	1
My child attends daycare 3 or more days per week		
**Group**	**Became Treatment compliant**	**Became Treatment Non-compliant**
RT	-	2
CRO	-	-
PT - RT	-	-
PT - CRO	-	-
I am happy with my child's current head shape		
**Group**	**Became Treatment compliant**	**Became Treatment Non-compliant**
RT	1	3
CRO	-	2
PT - RT	-	1
PT - CRO	1	1
